# Molecular mechanisms of mammalian sperm capacitation, and its regulation by sodium‐dependent secondary active transporters

**DOI:** 10.1002/rmb2.12614

**Published:** 2024-10-16

**Authors:** Gen L. Takei

**Affiliations:** ^1^ Department of Pharmacology and Toxicology Dokkyo Medical University Tochigi Japan

**Keywords:** intracellular pH, K^+^, Na^+^, secondary active transporter, sperm capacitation

## Abstract

**Background:**

Mammalian spermatozoa have to be “capacitated” to be fertilization‐competent. Capacitation is a collective term for the physiological and biochemical changes in spermatozoa that occur within the female body. However, the regulatory mechanisms underlying capacitation have not been fully elucidated.

**Methods:**

Previously published papers on capacitation, especially from the perspective of ions/channels/transporters, were extracted and summarized.

**Results:**

Capacitation can be divided into two processes: earlier events (membrane potential hyperpolarization, intracellular pH rise, intracellular Ca^2+^ rise, etc.) and two major later events: hyperactivation and the acrosome reaction. Earlier events are closely interconnected with each other. Various channels/transporters are involved in the regulation of them, which ultimately lead to the later events. Manipulating the extracellular K^+^ concentration based on the oviductal concentration modifies membrane potential; however, the later events and fertilization are not affected, suggesting the uninvolvement of membrane potential in capacitation. Hyperpolarization is a highly conserved phenomenon among mammalian species, indicating its importance in capacitation. Therefore, the physiological importance of hyperpolarization apart from membrane potential is suggested.

**Conclusion:**

The hypotheses are (1) hyperpolarizing Na^+^ dynamics (decrease in intracellular Na^+^) and Na^+^‐driven secondary active transporters play a vital role in capacitation and (2) the sperm‐specific potassium channel Slo3 is involved in volume and/or morphological regulation.

## 1 INTRODUCTION

An increasing number of people are suffering from infertility and subfertility, probably because of the tendency to marry later than in developed countries. Generally, older men have worse semen parameters and reproductive success.^1^ This trend has led to a decrease in the population, which is a serious problem for the maintenance of the nation and society. Excluding the effects of migration, it is predicted that many countries will experience a population decline of more than 50% between 2017 and 2100.^2^, ^3^ Infertility is experienced by an estimated 48.5 million couples worldwide.^4^ In Japan, the latest report (2021) showed that both assisted reproductive technology (ART) and live births resulting from ART are almost consistently increasing, despite the COVID‐19 pandemic.^5^ Thus, the demand for ART in the treatment of infertility is a matter of concern.

The first reproducible in vitro fertilization (IVF) method was achieved in the 1950s with rabbits.^6^, ^7^ However, the spermatozoa used in these studies were collected from the female reproductive tract after copulation, which is not applicable to human infertility treatment. The study by Yanagimachi and Chang, in which IVF was completely achieved outside the female body,^8^ opened the door for the birth of Louise Brown, the first test‐tube baby,^9^ and for today's ART. This work was groundbreaking because “capacitation” was realized in vitro in a defined medium (TC 199). Capacitation is a collective term used to describe the physiological and biochemical changes necessary for spermatozoa to acquire fertilization competency. Capacitation was first described almost simultaneously by the two researchers.^10^, ^11^ They found that spermatozoa acquire fertilization capacity when they reside in the female reproductive tract for a certain period, and Austin termed this phenomenon as “capacitation” to describe this maturational process.^12^

**FIGURE 1:**
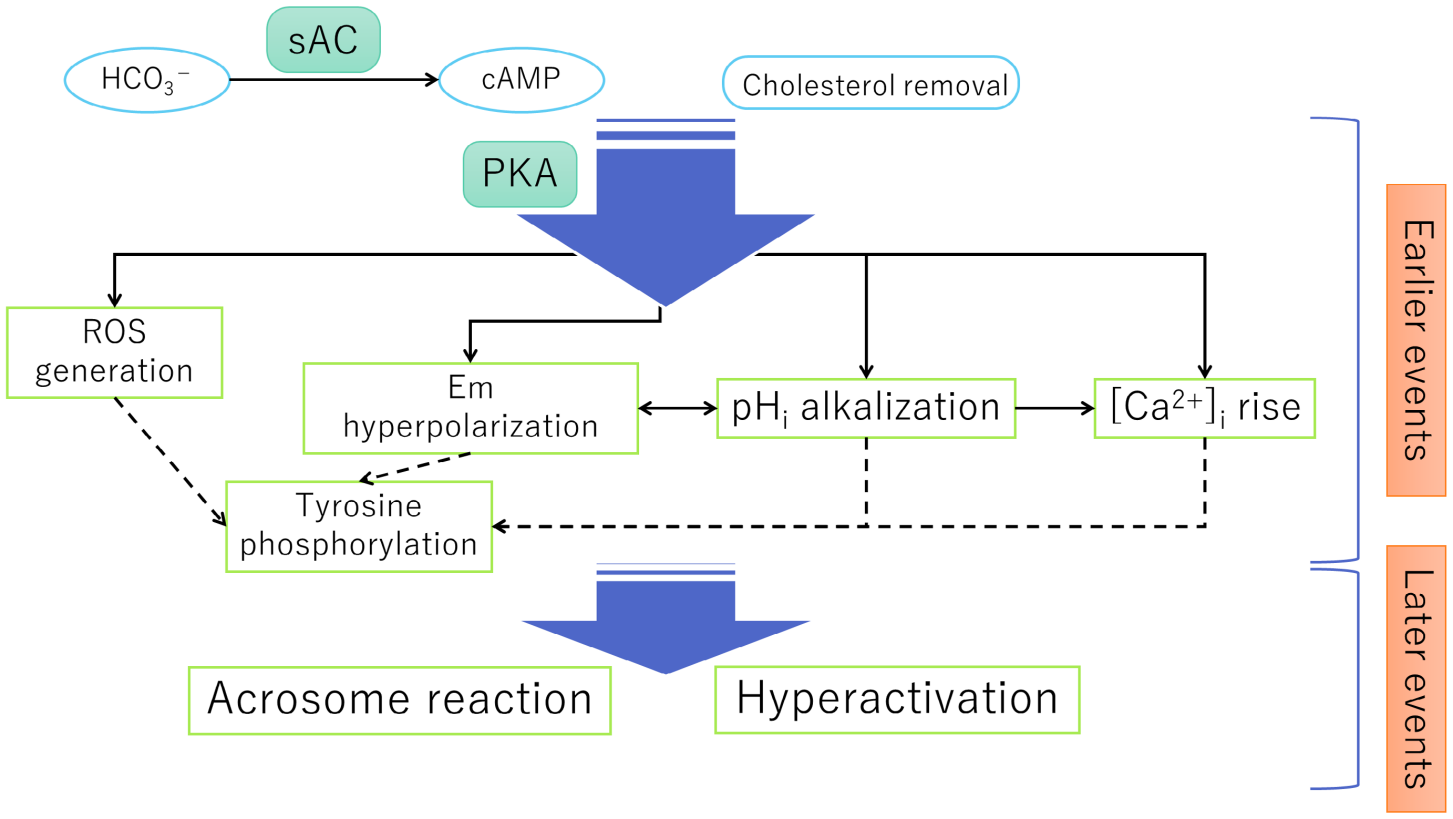
An overview of the major capacitation‐associated events. The major capacitation‐associated events are summarized. These events were divided into earlier and later capacitation events. Earlier events, which include ROS generation, hyperpolarization of membrane potential (Em), intracellular pH (pH_i_) alkalization, rise in intracellular Ca^2+^ ([Ca^2+^]_i_), and tyrosine phosphorylation of flagellar proteins, ultimately lead to two major later events: acrosome reaction and hyperactivation. All these capacitation‐associated events are virtually regulated downstream of the cAMP/PKA pathways, which are triggered by HCO_3_^−^ exposure and activation of sAC, as well as cholesterol removal from the plasma membrane. cAMP, cyclic adenosine monophosphate; PKA, protein kinase A; ROS, reactive oxygen species; sAC, soluble adenylate cyclase.

Thereafter, various capacitation‐associated changes have been described, such as (1) hyperactivation of flagellar motility, (2) acrosome reaction (AR), (3) hyperpolarization of the plasma membrane potential, (4) intracellular alkalization, (5) increase in intracellular Ca^2+^ and oscillation, (6) tyrosine phosphorylation of flagellar proteins, and (7) generation of reactive oxygen species (ROS). These changes are closely interconnected, and these events can be divided into two processes, earlier (or first) events that lead to later two major events of capacitation: hyperactivation and AR.^13^, ^14^ (Figure 1). All these capacitation‐associated events are regulated downstream of phosphorylation‐mediated signaling pathways: cyclic adenosine monophosphate (cAMP)/protein kinase A (PKA) pathways^13^, ^15^ (Figure 1) and removal of decapacitation factors^16^ followed by cholesterol efflux from the plasma membrane with the aid of a cholesterol acceptor (usually bovine serum albumin).^17^

Capacitation is triggered when spermatozoa are exposed to HCO_3_^−^ in the seminal fluid upon ejaculation, which directly activates soluble adenylate cyclase (sAC, also known as ADCY10).^18^, ^19^, ^20^ Direct activation of cAMP synthesis by HCO_3_^−^ was revealed in studies using porcine spermatozoa,^19^ followed by the discovery of sAC and its conserved function as a bicarbonate sensor.^18^, ^20^ Synthesis of cAMP by sAC activates PKA, which phosphorylates flagellar proteins.^21^ Currently, the importance of the cAMP/PKA pathway in sperm capacitation is widely supported by various gene‐modified animal models, such as sAC knockout mice,^22^ knockout mice of the sperm‐specific catalytic subunit of PKA^23^ (see review by Visconti [24]). Virtually all the above‐mentioned capacitation‐associated events are regulated downstream of the cAMP/PKA pathways, except that ROS generation was reported to be independent of the cAMP/PKA pathway in mouse spermatozoa.^25^

This review introduces the importance of the above‐mentioned capacitation‐associated events in fertilization and their molecular mechanisms based on the literature. Next, updated insights into capacitation with respect to the extracellular ionic milieu and channels/transporters are discussed, mainly based on our recent research using hamsters. Finally, hypotheses regarding the roles of Na^+^‐dependent secondary active transporters and the K^+^ channel (Slo3) are proposed.

## 2 CAPACITATION‐ASSOCIATED EVENTS AND THEIR PHYSIOLOGICAL IMPORTANCE

### 2.1 Hyperactivation

Yanagimachi observed that capacitated spermatozoa show very vigorous whiplash‐like motility with increased tail bending.^26^ Later, Yanagimachi named this sperm movement “hyperactivation.”^27^ This characteristic flagellar motility was first recognized in hamster spermatozoa and was found to be a universal phenomenon among mammalian species, such as humans,^28^, ^29^ mice,^30^ rats,^31^ bulls,^32^ pigs,^33^ guinea pigs,^34^ Chinese hamsters,^35^ and rabbits.^36^

Several physiological roles of sperm hyperactivation have been reported: (1) progression in the mucus environment in the oviduct^37^; (2) penetration of the zona pellucida (ZP), an extracellular matrix surrounding oocytes^38^, ^39^; and (3) release of spermatozoa with heads firmly attached to the reservoir on the surface of the isthmus of the oviduct.^40^, ^41^, ^42^, ^43^ Recently, it was shown that adovarian peristalsis causes the active fluid flow to transport spermatozoa toward the ovulated cumulus–oocyte complex in mice.^44^ Because the flow by peristalsis is much stronger than the swimming force of the spermatozoa, the role of hyperactivation in the progression of the mucus environment is less likely, and the release of spermatozoa from the reservoir of the isthmic epithelium to be transported by the flow is more likely.

The physiological importance of hyperactivation was supported by the studies using knockout mice of the sperm‐specific Ca^2+^ channel CatSper. Genetic deletion of CatSper‐α subunits (CatSper 1–4),^45^, ^46^, ^47^, ^48^, ^49^ or auxiliary subunits^50^, ^51^ caused impaired hyperactivation and infertility. Basal motility was also substantially altered in these gene‐modified animals; thus, we cannot exclude the possibility that the infertile phenotype of these animals was owing to basal motility defects, not hyperactivation defects. In humans, a considerable correlation between hyperactivation rate and success in IVF has been reported in several studies.^52^, ^53^, ^54^ Furthermore, treatments that enhance hyperactivation, such as transient exposure to Ca^2+^ ionophores^55^ or transient deprivation of energy nutrients and replenishment,^56^ improve the fertilization rate and development of fertilized embryos. These findings highlight the physiological importance of hyperactivation during fertilization both in vivo and in vitro. Furthermore, hyperactivation is an attractive target for ART development.

### 2.2 Acrosome reaction

The acrosome is a highly organized, cap‐like, membrane‐bound structure that covers the anterior part of the sperm head. The structures of the acrosome are apparently different among species but share common features: the acrosome consists of an acrosomal cap that covers the anterior region of the head and the equatorial segment, which is in the posterior region.^57^

The AR, which occurs on the acrosome of the sperm head, was first documented in echinoderm (sea urchin and starfish) spermatozoa and shows a filament‐like protrusion on the acrosome upon contact with egg water.^58^, ^59^ Six years later, AR was described in mammalian spermatozoa, in which the loss of the acrosome was observed in spermatozoa collected from the female reproductive tract shortly after coitus in rodents (golden hamster, guinea pig, Libyan jird, and Chinese hamster).^60^ AR has been observed in a diverse array of animal species, from invertebrates to humans. However, this review focuses on mammalian AR because the AR considerably varies among species (see review by Hirohashi and Yanagimachi [61]).

Because only spermatozoa that have completed AR can penetrate the ZP, and only spermatozoa can bind and fuse with eggs, the physiological role of AR is to render spermatozoa penetration and fusion competent. Calcium influx via Ca^2+^ channels, such as the voltage‐dependent Cav2.3^62^ and TRPC channels,^63^ is required to induce AR. Calcium influx via such channels induces the fusion of the outer acrosomal and plasma membranes, mediated by soluble *N*‐ethylmaleimide‐sensitive factor attachment protein receptor, which is presumably regulated by fer‐1like 5 (FER1L5).^64^ Acrosomal membrane fusion releases acrosomal hydrolytic enzymes, such as acrosin, hyaluronidase, and phospholipase A2, and relocates IZUMO, which is necessary for sperm–egg fusion.^65^, ^66^ The necessity for proteolyzing enzymes, such as acrosin, varies among species. Studies using acrosin knockout animal models revealed that acrosin is not essential for fertilization in mice,^67^ disruption of acrosin moderately impaired fertility in rats,^68^ and acrosin is essential for fertilization in hamsters.^69^ In addition, a nonsense mutation in the acrosin gene causes total fertilization failure in humans.^70^ The reason for these differences is not known, but it seems that acrosin facilitates the penetration of the ZP or the dispersal of cumulus cells.

Several substances have been identified that trigger AR. In vitro studies have shown that components of the ZP (e.g., ZP1, ZP2, and ZP3), progesterone,^71^ and neurotransmitters^72^ can induce AR in mice, humans, and hamsters. Based on these facts, it was previously thought that spermatozoa begin AR in contact with the ZP.^73^ In 2011, however, it was found that most fertilizing spermatozoa are already acrosome‐reacted before contact with the ZP.^74^ Moreover, mouse spermatozoa in vivo begin AR before reaching the ampulla of the oviduct, where fertilization occurs,^75^, ^76^, ^77^ and acrosome‐reacted spermatozoa collected from the perivitelline space can penetrate and fertilize other eggs.^78^ Considering that most mammalian spermatozoa can spontaneously undergo AR, these facts suggest that ligand‐mediated AR‐triggering mechanisms are merely auxiliary mechanisms and are not essential for fertilization.

### 2.3 Hyperpolarization of the sperm membrane potential

The potential of the sperm plasma membrane (Em) is substantially decreased (hyperpolarized) when spermatozoa are capacitated in vitro. This phenomenon was first described in mice and bulls,^79^ and has been shown to be a universal phenomenon among mammalian species (e.g., horses,^80^ humans,^81^ hamsters,^82^ and bats^83^). Hyperpolarization is associated with hyperactivation, AR, and fertility, and positive correlations between the hyperpolarization rate and hyperactivation, AR, and IVF rate have been observed in humans and mice.^84^, ^85^, ^86^, ^87^ Conversely, depolarization of the sperm membrane potential is associated with subfertility in patients undergoing IVF or intracytoplasmic sperm injection.^88^ Although these facts indicate the importance of hyperpolarization in sperm physiology, we should bear in mind that only a few pieces of evidence supporting a causal relationship between Em hyperpolarization and hyperactivation, AR, and IVF have been documented.^87^

The involvement of several channels and transporters in hyperpolarization has been investigated (Table 1), particularly in human and mouse models. In particular, the sperm‐specific K^+^ channel Slo3 plays a remarkable role in the capacitation‐associated hyperpolarization of the Em (see review by Lyon et al. [100]). Spermatozoa from Slo3 knockout mice exhibit depolarized Em, the absence of capacitation‐associated hyperpolarization, and infertility.^89^ Although Slo1, a ubiquitous paralog of Slo3, has been reported to be the principal K^+^ channel in human spermatozoa,^101^ studies using cultured cells heterologously expressing human Slo3 showed that Slo3 is the sole K^+^ channel that contributes to hyperpolarization in human spermatozoa.^102^

**TABLE 1:** Channels and transporters involved in hyperpolarization.

Gene name	Species	Experimental design	Phenotype	Reference
Slo3	Mouse	Knockout model, pharmacological inhibition	Depolarized Em, infertility, impaired motility, AR defect	[89, 90]
ENaC	Mouse	Pharmacological inhibition	Depolarized Em	[91]
CFTR	Mouse, human	Pharmacological inhibition	Depolarized Em, AR defect	[85, 93]
NBCe1 or NBCe2	Mouse, human	Pharmacological inhibition	Depolarized Em, phosphorylation defect	[85, 94]
Na^+^/K^+^ ATPase a4 subunit	Mouse, human, rat, hamster	Knockout model, pharmacological inhibition	Infertility, impaired motility/hyperactivation, depolarized Em	[95, 96, 97, 98, 99]

The participation of other channels/transporters in capacitation‐associated hyperpolarization processes has also been reported, although some studies are contradictory: closure of the epithelial Na^+^ channel (ENaC) and the subsequent decrease in intracellular Na^+^ concentration contribute to the hyperpolarization in mice,^91^, ^92^ although Chávez et al. reported that only a small change in Na^+^ permeability upon capacitation was observed compared to the large change in K^+^ permeability.^103^ In human and mouse spermatozoa, the involvement of the cystic fibrosis transmembrane conductance regulator (CFTR) in hyperpolarization by suppressing ENaC activity has been suggested, although the suggested roles of CFTR are different: Cl^−^ flux via the CFTR is crucial in mice, whereas transport of HCO_3_^−^ by CFTR is important in humans.^85^, ^93^ In mouse and human spermatozoa, HCO_3_^−^ transport via electrogenic Na^+^/HCO_3_^−^ cotransporter (NBCe1 or NBCe2) is also reportedly involved in the hyperpolarization process.^85^, ^94^

Na^+^/K^+^ ATPase (NKA) pump plays a crucial role in the regulation of sperm Em; its involvement is discussed in Section 3.

### 2.4 Intracellular alkalization

Alkalization of intracellular pH (pH_i_) in capacitated spermatozoa has been observed in mice,^104^ humans,^105^, ^106^ and bulls.^107^, ^108^ Intraacrosomal alkalization has also been observed in humans^109^ and mice,^110^ and has been implicated in AR.^111^, ^112^ Bull sperm hyperactivation has also been shown to be promoted by an increase in pH_i_.^113^ Moreover, intracellular alkalization directly activates the CatSper channel.^113^, ^114^ These facts highlight the importance of pH_i_ regulation in sperm physiology, although some investigators have raised questions regarding the physiological importance of the alkalization process in capacitation.^84^, ^115^

Although intracellular alkalization is an important event, the precise molecular mechanisms that regulate pH_i_ are still not fully elucidated (Table 2). In human spermatozoa, the voltage‐dependent proton channel (Hv1), which mediates zinc‐sensitive, depolarization‐dependent selective H^+^ currents,^132^, ^133^ plays a crucial role in pH_i_ regulation.^121^ Involvement of the Hv1 has also been suggested in bovine and porcine spermatozoa.^122^, ^123^ However, the Hv1 is absent in mouse spermatozoa,^121^ and Hv1‐deficient mice are fertile.^134^

**TABLE 2:** Channels and transporters involved in pHi regulation.

Gene name	Species	Experimental design	Phenotype	Reference
(s)NHE	Mouse, human	Knockout model, Pharmacological inhibition, semen analysis of mutated patients	Infertility, impaired cAMP pathway, impaired motility	[116, 117, 118, 119, 120]
Hv1	Human, bull, pig but not in mouse	Pharmacological activation/inhibition, patch clamp	Impaired motility, enhanced hyperactivation and capacitation	[121, 122, 123]
SLC4 family transporters (NDCBE, NBC)	Mouse, bull	Pharmacological inhibition	Inhibition of pH_i_ alkalization, impaired AR, no increase of tyrosine phosphorylation	[85, 94, 104]
CFTR/SLC26A3/SLC26A8 complex	Human, mouse Guinea pig	Pharmacological inhibition, elimination of Cl^−^, knockout model	Infertility, pH_i_ acidification, impaired hyperactivation, impaired AR, Em depolarization	[124, 125, 126, 127, 128, 129]
Carbonic anhydrase II and IV	Mouse	Knockout model, Pharmacological inhibition	Subfertility, impaired motility	[130, 131]

The expression and function of several Na^+^/H^+^ exchangers (NHE) have been reported, but more detailed information about the NHE is provided in Section 4.

The involvement of SLC4 family transporters (Na^+^‐dependent Cl^−^/HCO_3_^−^ exchanger^104^ and Na^+^/HCO_3_^−^ cotransporter (NBC)^94^) has also been suggested, but detailed information is discussed in Section 4.

CFTR^135^ and its interaction with SLC26 transporters (SLC26A3, SLC26A6, and SLC26A8)^124^, ^125^, ^126^ have also been suggested to be involved in pH_i_ regulation. SLC26A8 is sperm‐specific, and its knockout results in sterility^136^; missense mutations in the SLC26A8 gene in humans cause sperm malformation and asthenozoospermia,^127^ both of which suggest the importance of this transporter in sperm function. Further studies showed the interaction of SLC26A8 with CFTR and suggested a role for this interaction in HCO_3_^−^ transport,^125^ which in turn increases pH_i_, although SLC26A8 do not transport HCO_3_^−^.^128^ SLC26A3 and SLC26A6 also form a complex with CFTR, which plays a role in capacitation via pH_i_ alkalization and/or Em hyperpolarization,^126^ but pharmacological experiments suggest that only SLC26A3 plays a role in capacitation. These results were consistent with those of a study using *SLC26A3‐* and *SLC26A6‐*deficient mice showing only *SLC26A3‐*deficient mice exhibited impaired fertility.^129^ The importance of CFTR has also been demonstrated in human spermatozoa^137^; however, its involvement in pH_i_ regulation was not explored. Taken together, the molecular complex of CFTR/SLC26A3/SLC26A8 has an important role in pH_i_ regulation via HCO_3_^−^ transport.

Carbonic anhydrase (CA) catalyzes the reversible hydration of CO_2_ to HCO_3_^−^, thereby contributing to HCO_3_^−^/pH_i_ homeostasis. Two isoforms of CA, CAII and CAIV, are expressed in mouse spermatozoa, and their genetic deletion or pharmacological inhibition leads to motility defects, resulting in subfertility.^130^, ^131^ Although basal pH_i_ levels were not altered in the double knockout of CAII and CAIV,^131^ this molecule may participate in the regulation of the intracellular CO_2_/HCO_3_^−^/H^+^ equilibrium, as suggested in a recent study.^115^

### 2.5 Intracellular Ca^2+^ regulation

Calcium is a prerequisite for the functions of various cell types, including spermatozoa.^138^ The elimination of Ca^2+^ from the media prevents hyperactivation and AR.^57^ Mean intracellular Ca^2+^ levels were elevated upon capacitation,^139^ and intracellular Ca^2+^ oscillated at the frequency of the flagellar beat.^140^ An increase in Ca^2+^ concentration in the acrosome triggers AR,^141^, ^142^ whereas an increase in the flagellum promotes hyperactivation.^113^, ^143^ These findings indicate the importance of Ca^2+^ signaling in sperm physiology.

Several channels/transporters that regulate intracellular Ca^2+^ levels have been identified. Among these, the CatSper channel plays a central role. As mentioned in Section 2.1, genetic deletions of CatSper subunits cause severe motility defects and infertility.^40^, ^45^, ^46^, ^47^, ^48^, ^49^, ^50^, ^51^ In addition, it was shown that the principal Ca^2+^ current measured in the sperm flagellum is mediated by CatSper.^114^ Furthermore, a direct connection between defective CatSper function and male infertility was shown in humans.^144^ These findings indicate that this molecule plays a pivotal role in sperm capacitation. For more detailed information, please refer to excellent reviews.^145^, ^146^

The involvement of the voltage‐dependent Ca^2+^ channels (CaV channels) has also been reported.^138^ Currents in CaV channels have been detected in sperm cells, and pharmacological inhibition of CaV channels inhibits AR (summarized in Darszon et al. [138]). However, knockout models of CaV 1.3,^147^ CaV2.2,^148^ CaV3.1, and CaV3.2^149^, ^150^ channels are fertile except that knockout of the pore‐forming α1E subunit of CaV 2.3 caused subfertility and AR defect.^62^ This suggests that although CaV channels are present and functional in sperm cells, most are dispensable for sperm function.

Cyclic nucleotide‐gated (CNG) channels are non‐selective cation channels that open upon binding to cAMP or cGMP.^151^ Although mouse spermatozoa are responsive to membrane‐permeable cGMP, and pharmacological inhibition of CNG channels decreases capacitation,^152^, ^153^ mice deficient in the A3 subunit of the CNG channel are fertile.^151^ This suggests that CNG channels are not essential for sperm capacitation, although they play a role in the chemotaxis of sea urchin^154^ and ascidian spermatozoa.^155^

Other Ca^2+^ channels, such as transient receptor potential channels (TRP channels; TRPV1,^156^ TRPM8,^157^ and TRPC2^63^), two‐pore channel 1, and Ca^2+^ release‐activated Ca^2+^ channels,^158^ are reportedly involved in the regulation of capacitation, but further studies are needed to determine whether they are necessary for capacitation or just have an auxiliary role in optimizing fertilization in vivo.

There are several excellent reviews on calcium and capacitation, so if the readers want to know this topic in more detail, please refer to them.^138^, ^145^, ^146^, ^159^, ^160^

### 2.6 Protein tyrosine phosphorylation

In 1995, it was first reported that the tyrosine residues of sperm proteins were strongly phosphorylated upon capacitation in studies using mouse spermatozoa.^161^, ^162^ Protein tyrosine phosphorylation is regulated by the cAMP/PKA pathway.^161^, ^162^ Subsequently, the intense phosphorylation of sperm proteins was found to be universal in humans,^163^ boars^164^ bulls,^165^ and hamsters.^166^ The molecular identity of tyrosine kinases has long been unknown. Finally, it was demonstrated using pharmacological and genetic approaches that the testis‐specific isoform of the non‐receptor protein tyrosine kinase FER (FERT) is the molecule responsible for capacitation‐associated tyrosine phosphorylation.^167^ Surprisingly, mice carrying a kinase‐inactivating mutation in FER (*Fer* ^*DR/DR*^) were fertile in vivo although fertilization in vitro was severely impaired.^167^ These findings indicate that capacitation‐associated tyrosine phosphorylation is not essential for fertilization in vivo. It was postulated that the functional role of protein tyrosine phosphorylation in vivo could be bypassed by yet undiscovered mechanisms, whereas there is a presumption that, at least in mice, the physiological role of tyrosine phosphorylation is to maximize the efficiency of sperm fertilization.^168^ Further studies are needed to determine whether this highly conserved phenomenon merely plays an auxiliary role or is crucial and thus is compensated by a yet to be discovered mechanism to ensure capacitation/fertilization.

### 2.7 Reactive oxygen species (ROS) generation

ROS is a collective name of radicals formed from diatomic oxygen, such as superoxide anions ($O_{2}^{\cdot -}$), hydrogen peroxide (H_2_O_2_), and hydroxyl radicals (O⋅H).^169^ ROS are highly toxic, as they damage DNA, proteins, and lipids, ultimately leading to apoptosis.^170^ However, the active generation of ROS by mammalian spermatozoa has been known as early as the 1940s.^171^, ^172^ At first, the harmful effects of ROS on sperm function and the negative correlation between them have been intensively studied (see the review by Aitken [170]). In 1993, however, de Lamirande and Gagnon reported that hyperactivation is dependent on $O_{2}^{\cdot -}$.^173^, ^174^ In addition, the elimination of H_2_O_2_ by catalase almost completely suppressed fertilization in vitro in mice,^25^ and catalase also blocked the penetration of human spermatozoa into hamster ova.^175^ Moreover, mild oxidative stress induced by ferrous ion promoters enhanced zona binding in humans^176^ and mice.^177^ ROS generation enhances tyrosine phosphorylation in humans,^175^, ^178^ rats,^179^ buffaloes,^180^ bulls,^181^ and horses,^182^ suggesting that the positive effects of ROS on capacitation/fertilization are achieved via tyrosine phosphorylation pathways. However, tyrosine phosphorylation is independent of ROS in mice,^25^ and the physiological importance of tyrosine phosphorylation is uncertain, as discussed above. Considering the conserved importance of ROS in capacitation, ROS may regulate capacitation through several pathways to exert positive effects.

Despite the abundance of studies supporting the importance of ROS in sperm physiology, the molecular identity of ROS‐generating molecules remains uncertain; NADPH oxidase 5 (NOX5) has been reported to be a major source of ROS in human,^183^ equine,^184^ and ram^185^ spermatozoa. However, Nox5 is absent in rodents.^186^ Other subtypes of Noxs, such as Nox2 and Nox4, have been reported to be involved in ROS generation in guinea pigs and mice.^187^ Enzymes other than Noxs, lipoxygenases,^188^, ^189^ and l‐amino acid oxidases,^190^ have also been proposed to be involved in sperm ROS generation. However, a conclusive study to determine the ROS‐generating molecules in spermatozoa has yet to be conducted.

## 3 THE EFFECTS OF OVIDUCTAL EXTRACELLULAR IONS ON HAMSTER SPERM CAPACITATION

As described above, capacitation induces many physiological and biochemical changes, and later events, such as hyperactivation and AR, are generally regulated downstream of earlier events. Our research group has been studying the regulatory mechanisms by which capacitation‐associated earlier events ultimately lead to later events. To elucidate sperm behavior in vivo, it is important to consider the environment in situ. Because capacitation/fertilization occurs in the oviduct,^191^ the ionic composition of the oviductal fluid was investigated^192^ (Table 3). For this study, hamsters were used as a model for the following reasons: (1) hamsters were the first animal model to achieve IVF; (2) the estrus cycle is very stable (4 days/cycle) and is easy to monitor by vaginal discharge; (3) short gestation period (16 days) and large litter size (4–12 pups); (4) spermatozoa have long and flexible flagella that allow easy recognition of hyperactivation and other motility changes; and (5) large acrosomes, which allow easy observation of acrosomal status.

**TABLE 3:** Na and K concentration of the oviductal fluid and the media used for IVF.

	Oviductal fluid (mEq/L)	mTALP (mM)	TYH (mM)	HTF (mM)
Na	158.79 ± 5.76	138.26	145.44	148.33
K	20.95 ± 1.87	2.68	5.97	5.06

*Note*: Concentrations of Na and K in the oviductal fluid and IVF media are summarized. The concentrations of elements were expressed in mEq/L in oviductal fluid, and mM in the media.

Determination of the ionic composition of oviductal fluid revealed that Na^+^ and K^+^ concentrations of the oviductal fluid (158.79 ± 5.76, 20.95 ± 1.87 mEq/L, respectively, Table 3) were considerably higher than those of capacitation/fertilization‐supporting medium, such as modified Tyrode's Albumin Lactate Pyruvate medium (mTALP; 138.26 mM Na^+^ and 2.68 mM K^+^, respectively), Toyoda–Yokoyama–Hosi medium (TYH medium; 145.44 mM Na^+^ and 5.97 mM K^+^),^193^ and human tubal fluid medium (HTF; 148.33 mM Na^+^ and 5.06 mM K^+^),^194^ (Table 3). Oviductal K^+^ concentration (~20 mM) seems unfavorable because such high concentrations of K^+^ normally depolarize Em and thus hinder capacitation/fertilization. Indeed, the depolarization of Em by high concentrations of K^+^ (>20 mM) suppressed ZP‐induced AR.^87^ Nevertheless, the high content of K^+^ in the oviduct fluid was not unique to hamsters but was also observed in mice and humans (17.8–28.6 mM K^+^ ^195^, ^196^, ^197^). The uterine fluid of the rats also contains a high concentration (37.4–37.5 mEq/L) of K^+^.^198^ Furthermore, some studies have reported the beneficial effects of high K^+^ concentrations on capacitation.^199^, ^200^ Therefore, I investigated the effect of the oviductal concentration of K^+^ on membrane potential and capacitation/fertilization in hamsters. The oviductal concentration of K^+^ (20 mM) considerably depolarized the Em compared to that in the presence of a conventional K^+^ concentration (2.68 mM). Em significantly decreased (hyperpolarized) after capacitation, even in the presence of oviductal concentrations of K^+^ compared to that before capacitation. However, the hyperpolarized Em value was significantly higher than that of the uncapacitated spermatozoa in the presence of a conventional K^+^ concentration (2.68 mM). Despite the increased (depolarized) Em caused by the oviductal concentration of K^+^, later events of capacitation (hyperactivation and AR) and IVF were not significantly affected by the oviductal K^+^ concentration.^82^ Since Em‐dependent events occur depending on the certain “value” (or a threshold) of Em, these facts indicate that the physiological importance of capacitation‐associated hyperpolarization of Em is not exerted in a voltage‐dependent manner. In this sense, hyperpolarization during capacitation can be used as a capacitation marker but is not a quantitative change; thus, hyperpolarization of the Em itself does not have physiological relevance. Considering these facts, the term “hyperpolarization” should be revised (e.g., decrease of Em) because the term “hyperpolarization” reminds us of the voltage‐dependent phenomena or certain threshold of Em. However, capacitation and fertilization actually occur independently of the Em value.

Determination of the ionic composition of the oviductal fluid revealed that the Na^+^ concentration was higher than that in the mTALP medium (Table 3). Thus, the effect of extracellular Na^+^ concentration on hyperactivation, a later event in capacitation, was investigated. An increase of extracellular Na^+^ concentration from 112.26 to 161.26 mM by changing the NaCl concentration from 75 to 150 mM significantly lowered intracellular Ca^2+^ and delayed the expression of hyperactivation.^192^ This effect of extracellular Na^+^ did not change when the extracellular osmolality of the medium was compensated for by mannitol. Furthermore, the Na^+^/Ca^2+^ exchanger (NCX)‐specific inhibitors SEA0400 and SN‐6 canceled the delay in hyperactivation by extracellular Na^+^. A previous study showed that hamster spermatozoa possess functional NCX1 and that its activity is downregulated upon capacitation, presumably by decreasing phosphatidylinositol 4,5‐bisphosphate (PIP_2_) levels.^201^, ^202^ These facts strongly suggest that NCX1 is a hyperactivation “brake,” and its downregulation is necessary to trigger hyperactivation.

The fact that Na^+^ and its secondary active transporter, NCX1, are involved in the regulation of capacitation/hyperactivation suggests the involvement of the primary active transporter, Na^+^/K^+^ ATPase (NKA), in sperm capacitation/hyperactivation. NKA plays a predominant role in regulating membrane potential and cellular sodium homeostasis. NKA consists of catalytic α subunit and auxiliary β subunit, and mammalian spermatozoa possess ubiquitous NKA α1 and sperm‐specific α4.^203^, ^204^, ^205^, ^206^ The importance of NKA α4 in sperm capacitation/hyperactivation has been reported in humans,^97^, ^206^ mice,^95^ rats,^98^, ^203^, ^204^, ^205^, ^207^ and bulls.^208^, ^209^ NKA α4 is more highly sensitive to ouabain, a well‐known cardiotonic steroid that specifically inhibits NKAs, than α1,^95^, ^97^, ^204^ namely, 10^−6^ M (1 μM) ouabain specifically inhibits NKA α4, whereas >10^−5^ M (10 μM) ouabain inhibits both NKA α1 and α4. Thus, I investigated the role of NKA α4 and α1 on sperm capacitation/hyperactivation utilizing this difference in the sensitivity to ouabain. When hamster spermatozoa were treated with 1 μM ouabain, hyperactivation was almost completely inhibited, whereas the percentage of motile spermatozoa was not affected.^96^ In contrast, >10 μM ouabain inhibit both hyperactivation and percentage of motile spermatozoa. A more detailed analysis of flagellar motility revealed that capacitated spermatozoa showed decreased flagellar beat frequency and increased flagellar bend angles compared to those of uncapacitated spermatozoa, which is a typical pattern of hyperactivated motility.^210^ In contrast, 1 μM ouabain inhibited such hyperactivation‐associated change in flagellar motility; both decreases in beat frequency and increase in bend angles were significantly suppressed by 1 μM ouabain. Moreover, the sliding velocity of the microtubule (rad/s) was indirectly assessed as a product of the beat frequency and bend angle to estimate the effect of ouabain on the activity of the motor protein dynein.^211^, ^212^ Inhibition of NKA α4 by 1 μM ouabain did not affect sliding velocity, suggesting that 1 μM ouabain specifically inhibited hyperactivation‐associated change of flagellar motility without inhibiting dynein activity. On the other hand, when spermatozoa were treated with >10 μM ouabain to inhibit both NKA α1 and α4, sliding velocity was substantially inhibited. These facts suggest that NKA α1 is necessary for the maintenance of flagellar motility, whereas NKA α4 is necessary for the change of flagellar motility associated with hyperactivation.^96^

## 4 Na^+^‐DRIVEN TRANSPORTERS AND SPERM CAPACITATION

As discussed above, Em is not important for the later events of sperm capacitation and fertilization. In contrast, Em hyperpolarization is a highly conserved phenomenon indicative of fertilization competency in human spermatozoa. This indicates that the changes in ionic dynamics that induce hyperpolarization are more important than those in Em itself. Among the ionic dynamics that induce hyperpolarization, the importance of Na^+^ dynamics in capacitation has been suggested for several reasons: (1) High concentrations of K^+^ had no significant effect on hyperactivation, AR, and IVF. (2) Sperm‐specific NKA is highly conserved among mammalian species, and its activity is indispensable for mammalian sperm capacitation/hyperactivation. (3) NKA activity is upregulated during capacitation in spermatozoa of several species (e.g., hamsters,^213^ rats,^214^ and bull^209^). (4) A decrease in intracellular Na^+^ levels occurs during capacitation (which is also indicative of Em hyperpolarization).^91^ (5) Such Na^+^ dynamics facilitate Na^+^‐dependent secondary active transporters (NDSAT), which in turn modify various physiological parameters (e.g., pH and intracellular Ca^2+^) of spermatozoa.

Among NDSATs, those regulating pH_i_ seem to play important roles downstream of capacitation‐associated Na^+^ dynamics because hyperpolarizing Na^+^ dynamics facilitate pH_i_ alkalization, which is another hallmark of capacitation, by NDSAT. Therefore, I propose that the upregulation of NDSAT, especially those regulating pH_i_ via hyperpolarizing Na^+^ dynamics, plays a crucial role in the regulation of later capacitation events. Several studies have shown a relationship between NKA and pH_i_ in rat^99^, ^207^ and human^116^ spermatozoa.

This section introduces the literature on mammalian sperm NDSAT and discusses its importance in sperm capacitation.

### 4.1 Na^+^/H^+^ exchangers (NHE)

The SLC9 gene family, which encodes the NHE, is a well‐known pH_i_ regulator in various cell types. NHE extrudes H^+^ from the cytosol in exchange for Na^+^ utilizing the electrochemical energy of the Na^+^ gradient and is a major pH_i_ regulator in various cell types. The expression of NHE1,^117^ NHE5,^207^ and sNHE (aka. NHE10)^118^, ^215^ and testis‐specific NHE11^216^ in spermatozoa have been reported. Because hyperpolarizing Na^+^ dynamics enhance the driving force of NHE, these NHE seem to play a role in sperm capacitation. Genetic deletion of sNHE causes infertility in mice,^118^ and mutations in SLC9C1 (sNHE) cause severe defects in human sperm motility and fertility,^119^ indicating the importance of this molecule in sperm function. However, in sNHE‐knockout mice, the motility defect phenotype was restored when spermatozoa were co‐incubated with a cAMP analog. A subsequent study by the same research group showed that sNHE‐null spermatozoa failed to express sAC, an essential molecule for mammalian sperm capacitation.^120^ Although this research group has also shown that sNHE has modest but consistent Na^+^–H^+^ exchange activity, these facts indicate that the infertility phenotype of sNHE‐knockout mice can be largely attributed to a lack of sAC. Studies using NHE inhibitors (e.g., ethyl‐isopropyl amiloride (EIPA), 5‐(*N,N*‐dimethyl)‐amiloride (DMA), and amiloride) have suggested the importance of NHE activity in the regulation of pH_i_ and sperm capacitation^116^, ^117^, ^217^; however, further studies are needed to identify the isoform of NHE responsible for pH_i_ regulation and sperm function.

### 4.2 Sodium bicarbonate cotransporter (NBC)

NBCs are encoded by the SLC4 family. The SLC4 family consists of 10 genes (*SLC4A1–5* and *SLC4A7–11*). Nine of these encode transporters that transport a bicarbonate ion (HCO_3_^−^) or an equivalent (CO_3_^2−^) across the plasma membrane, and five of them transport HCO_3_^−^ in a Na^+^‐dependent manner (NBCe1, NBCe2, NBCn1, NBCn2, NDCBE; reviewed by Romero et al. [218]). NBCe1 and NBCe2 (SLC 4A4 and 4A5) are electrogenic, whereas NBCn1, NBCn2, and NDCBE (SLC 4A7, 4A10, and 4A8, respectively) are electroneutral. Most SLC4 family transporters play an important role in acid–base homeostasis in many cell types and are functionally inhibited by 4,4′‐diisothiocyanatostilbene‐2,2′‐disulfonate (DIDS).

A study using mouse spermatozoa as a model showed that recovery after acid‐loading, as well as capacitation‐associated intracellular alkalization, were dependent on the extracellular Na^+^, Cl⁻, and HCO_3_^−^, and this alkalization was inhibited by DIDS.^104^ This suggests that the intracellular alkalization in mouse spermatozoa is mediated by sodium‐dependent chloride bicarbonate exchanger (NDCBE).^104^ The expression of NDCBE mRNA in the testis has been confirmed in rats,^219^ but the presence of NDCBE protein has not been shown to date. Demarco et al. demonstrated that DIDS‐sensitive and electrogenic Na^+^/HCO_3_^−^ cotransport activity is present in mouse spermatozoa and is associated with Em hyperpolarization, tyrosine phosphorylation, and ZP‐induced AR.^94^ NBCe1 expression in rat spermatozoa was confirmed using western blotting.^220^ A study using S0859, an inhibitor of pan‐cardiac NBCs,^221^ reported that NBC and ENaC are essential for the CFTR‐dependent activation of cAMP/PKA signaling and Em hyperpolarization in human spermatozoa, although this study was silent on the NBC isoforms responsible for this function.^85^ In humans, NBCe2 and NBCn2 mRNA have been detected in the testes,^219^ but neither the existence of the protein in spermatozoa nor its function has yet been explored. To the best of our knowledge, no information is available regarding other NBCs (NBCe2 and NBCn1).

Because HCO_3_^−^ plays a key role in capacitation and capacitation‐associated Na^+^ dynamics upregulate the driving force of HCO_3_^−^ transport by NBCs, the involvement of these NBCs in sperm capacitation is likely. However, further studies using other approaches, such as genetic deletion models or pharmacological approaches using more specific inhibitors, are required.

### 4.3 Na^+^/Ca^2+^ exchangers (NCX)

Na^+^/Ca^2+^ exchangers (NCX) are encoded by the SLC8 family. In mammals, there are three SLC family genes (*SLC8A1*, *SLC8A2*, and *SLC8A3*, which encode NCX1, 2, and 3, respectively), and several splice variants that are expressed in a tissue‐specific manner.^222^ NCX transports Ca^2+^ in exchange for Na^+^ utilizing the electrochemical gradient of Na^+^, and it mediates Ca^2+^ fluxes either in the Ca^2+^ efflux (forward) or Ca^2+^ influx (reverse) mode, depending on the gradient of Na^+^ across the plasma membrane.^222^ Distinct NCX isoforms contribute to excitation–contraction coupling of the heart, long‐term potentiation of the brain and learning, blood pressure regulation, immune response, neurotransmitter and insulin secretion, and mitochondrial bioenergetics. Knockout of NCX1^223^ and NCX2^224^ causes embryonic lethality, whereas NCX3 knockout mice exhibit skeletal muscle fiber necrosis and defective neuromuscular transmission,^225^ highlighting the importance of NCX molecules in the physiological functions of the whole body.

In mammalian spermatozoa, NCX‐like activity was reported as early as the 1980s by Bradley and Forrester, where they showed that both Na^+^‐dependent Ca^2+^ influx and Na^+^‐dependent Ca^2+^ efflux in ram spermatozoa.^226^ Wennemuth et al. also observed a Na^+^‐dependent intracellular Ca^2+^ clearance mechanism using mouse spermatozoa.^227^ However, these studies were silent on the physiological role (e.g., motility and capacitation) of Na^+^/Ca^2+^ exchange activity. In 2001, a pharmacological study using 2′,4′‐dichlorobenzamil as an NCX inhibitor suggested that NCX is involved in the maintenance of sperm motility.^228^ A later study using 3′,4′‐dichlorobenzamil hydrochloride, bepridil, and KB‐R7943 as NCX inhibitors also suggested that NCX is necessary for Ca^2+^ homeostasis and maintenance of motility in human spermatozoa.^229^ However, the inhibitors used in these studies are less specific than SEA0400 and SN‐6, which were used in our previous studies, and they inhibit several channels/transporters, receptors, and signaling molecules (e.g., CNG channels, store‐operated Ca^2+^ entry, L‐type Ca^2+^ channels, Na^+^ channels, ryanodine receptors type 1 and type 2, NMDA receptors, and calmodulin) in the micromolar range.^230^, ^231^, ^232^, ^233^ In addition, the NCX isoform expressed in spermatozoa is unknown. In this sense, our previous studies showing that NCX1 is present in hamster spermatozoa and functions as a “brake” of hyperactivation are the first studies to clearly show the presence and physiological function of NCXs in mammalian spermatozoa.^192^, ^201^

Capacitation should reinforce NCX1 function to suppress hyperactivation because capacitation‐associated Na^+^ dynamics facilitate NDSAT. This seems to be the reason why hamster spermatozoa downregulate NCX1 activity by a decrease in membrane PIP_2_ levels.^201^ Recently, voltage‐sensing phosphatase (VSP) has been shown to regulate PIP_2_ levels and distribution in the mouse sperm plasma membrane.^234^ Therefore, VSP may be responsible for regulating NCX1 activity in hamster spermatozoa. Further studies are required to fully elucidate the NCX1‐mediated regulatory mechanism of hyperactivation.

### 4.4 Other NDSATs

#### 4.4.1 Na^+^–K^+^–Cl^−^ cotransporter (NKCC)

NKCC1 and NKCC2, which are encoded by *SLC12A2* and *SLC12A1*, respectively, transport Cl^−^ together with Na^+^ and K^+^.^235^ In mouse spermatozoa, NKCC1 was detected by western blotting, and its specific inhibitors, furosemide and bumetanide, inhibited capacitation‐associated tyrosine phosphorylation and ZP‐induced AR.^236^ NKCC may regulate these capacitation‐associated events via Cl^−^ transport, although the exact role of Cl^−^ transport has not yet been determined.

#### 4.4.2 Sodium‐dependent glucose transporter (SGLT)

SGLT encoded by the *SLC5* family of genes is a sodium symporter that facilitates glucose transport across the plasma membrane. Two types of SGLTs are expressed in different tissues: SGLT1 in the small intestine and SGLT2 in the renal proximal convoluted tubules, both of which incorporate glucose in a Na^+^‐dependent manner.^237^

In spermatozoa, it was reported that spermatozoa of NKA α4 deficient mice exhibit lower glucose uptake.^238^ In addition, mouse spermatozoa possess SGLT1 but not SGLT2, and the SGLT‐specific inhibitor, phlorizin, impairs sperm metabolism and motility.^238^ Moreover, the genetic deletion of *SGLT1* caused a mild but significant defect in sperm motility.^239^ Studies using mice as a model have shown that spermatozoa upregulate glucose consumption^240^ and glycolytic^241^ and oxidative phosphorylation flux^242^ upon capacitation, presumably to fulfill the increased energy demand during capacitation. Thus, the upregulation of SGLT1 by capacitation‐associated Na^+^ dynamics appears to facilitate the upregulation of metabolism to support capacitation.

#### 4.4.3 Taurine transporter (TauT)

The taurine transporter (TauT), which is encoded by *SLC6A6*, transports taurine in a Na^+^‐ and Cl^−^‐dependent manner.^243^ Mice with disrupted SLC6A6 exhibit retinal degradation and severe defects in female fertility, highlighting the physiological significance of TauT.^244^

Recently, TauT was shown to transport hypotaurine, a taurine precursor.^245^ Hypotaurine is abundant in the oviductal fluid (~1.525 mM)^246^, ^247^, ^248^ and has beneficial effects on sperm physiology in hamsters^248^, ^249^, ^250^ and other mammals,^251^ although there are interspecies variations in hypotaurine effectiveness; hypotaurine is necessary for the maintenance of motility in hamster spermatozoa while it is not essential for human and bovine spermatozoa. Hypotaurine prevents membrane lipid peroxidation.^251^ Therefore, the mTALP medium used for hamster sperm experiments was supplemented with hypotaurine.^252^ Finally, a recent study showed that hamster spermatozoa incorporate and concentrate hypotaurine via an action of TauT utilizing an electrochemical gradient of Na^+^ and Cl^−^, to protect themselves against ROS.^253^ The importance of TauT has also been suggested in human and mouse spermatozoa; a decrease in TauT expression is associated with teratozoospermia in humans, and decreased levels of TauT/taurine in spermatozoa lead to developmental defects in embryos and recurrent pregnancy loss.^254^ Therefore, TauT and taurine/hypotaurine are promising candidates for improving ART.

## 5 POSSIBLE ROLE OF K^+^ CHANNEL SLO3

As discussed above, it has been suggested that the membrane potential is independent of capacitation/fertilization and that Em hyperpolarization is a result of changes in Na^+^ dynamics, but not K^+^, which is necessary for later capacitation events. However, several studies have demonstrated the importance of the K^+^ channel Slo3 for capacitation/fertilization.^89^, ^90^, ^255^ Thus, this section reviews the literature regarding the physiological function of Slo3 in detail and proposes a hypothesis regarding the physiological role of Slo3 in capacitation/fertilization.

As mentioned above, genetic and pharmacological approaches have been employed to elucidate the physiological functions of Slo3. Both Slo3 inhibition and genetic deletion of Slo3 inhibit overall capacitation–associated events (i.e., hyperpolarization of Em, hyperactivation, AR, and IVF).^89^, ^90^, ^255^ Further studies were conducted to elucidate the mechanism of Slo3 channel regulation of capacitation/fertilization and revealed the association between Slo3 and CatSper: Slo3 activation leads to the activation of the CatSper channel via hyperpolarization of Em to express hyperactivated motility.^90^, ^234^, ^256^ Because CatSper plays a crucial role in capacitation, especially hyperactivation, this hypothesis seems likely. When the phenotypes of spermatozoa of Slo3 null mice were more closely compared with those of CatSper null mice, Slo3 null spermatozoa showed more severe defects than those of CatSper null spermatozoa; CatSper null spermatozoa exhibited normal AR and were able to fertilize ZP‐free eggs^45^ whereas Slo3 null spermatozoa were unable to undergo AR and fertilize ZP‐free eggs.^89^ If the main role of Slo3 is to regulate CatSper activity, Slo3‐null spermatozoa should be able to undergo AR and fertilize ZP‐free eggs, as observed in CatSper‐null spermatozoa. Therefore, a crucial function of Slo3 is not to regulate CatSper and hyperactivation, but the existence of other indispensable functions of Slo3 has been suggested, as recently suggested,^257^ despite the substantial interconnection between Slo3 and CatSper.

Zeng et al. reported that Slo3 null spermatozoa showed morphological abnormalities owing to osmoregulatory defects, especially when exposed to hypotonic conditions.^255^ In addition, Lv et al. reported that a homozygous mutation in Slo3 leads to asthenoteratozoospermia owing to malformations of the acrosome and mitochondrial sheath in human males.^258^ K^+^ channels play a substantial role in regulating the cell volume via K^+^ efflux upon exposure to hypotonic conditions.^259^ This volume‐regulating process is known as the regulatory volume decrease (RVD). In sperm cells, RVD defects result in hindrance of mucus penetration in humans and failure of uterotubal passage in mice.^260^ In addition, broad‐spectrum K^+^ channel inhibitors, such as clofilium, which also inhibits Slo3,^255^ impair sperm RVD.^260^ Thus, it was hypothesized that Slo3 is involved in RVD and that severe fertilization defects in Slo3‐null spermatozoa are attributed to morphological abnormalities caused by impaired volume regulation. Such morphological defects in the acrosome and the subsequent inability to induce the regulated AR are likely the main reasons for infertility in Slo3‐null spermatozoa. The function of Slo3 in the regulation of CatSper activity may be an auxiliary function that optimizes fertilization efficacy. Further studies are needed to validate this hypothesis, but I believe that it is worth investigating.

## 6 CONCLUSIONS

**FIGURE 2:**
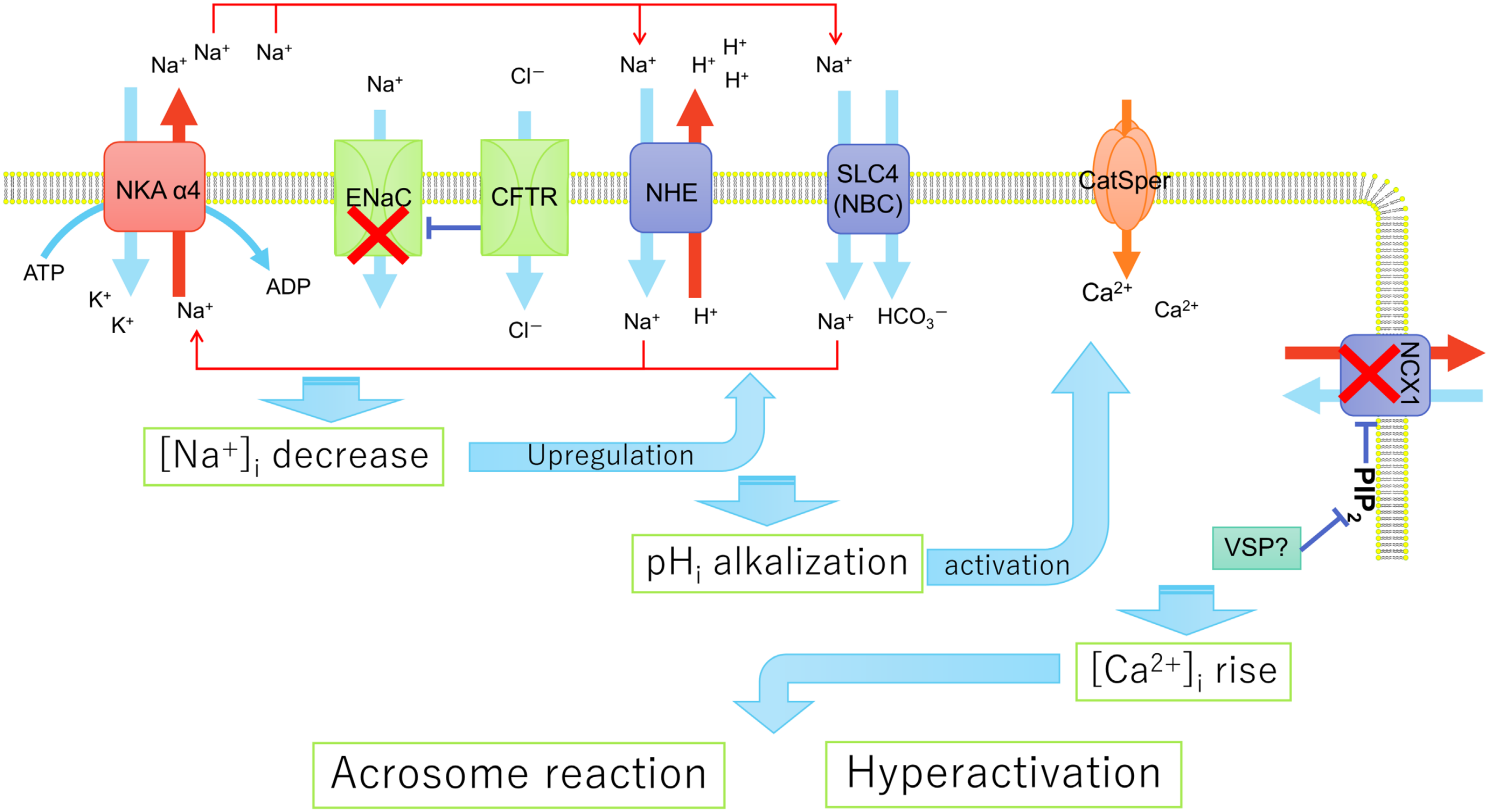
Schematic model of capacitation regulated by NDSAT. The proposed hypotheses regarding the regulatory mechanisms of capacitation from the aspect of ions/transporters/channels are presented. In this model, transporters or molecules independent of Na^+^ or its regulation (e.g., SCL26A3/8, CA, Hv1) were omitted to simplify the hypothesis. CFTR, cystic fibrosis transmembrane conductance regulator; ENaC, epithelial Na^+^ channel; NCX1, Na^+^/Ca^2+^ exchanger 1; NHE, Na^+^/H^+^ exchanger, several isoforms of NHE (NHE1, NHE5, sNHE, and NHE11) are shown to be present in spermatozoa; NKA α4, Na^+^/K^+^ ATPase subunit α4; SLC4 (NBC), solute carrier family 4 (Na^+^ bicarbonate co‐transporter), the isoform present in spermatozoa, remains to be identified; VSP, voltage sensor containing phosphatase.

Capacitation can be divided into two parts, earlier events and later major two events, AR and hyperactivation. Earlier events include hyperpolarization, pH_i_ alkalization, intracellular Ca^2+^ regulation, protein tyrosine phosphorylation, and ROS generation, and various channels/transporters are involved in their regulation which ultimately lead to the latter two events. Finally, this study proposed two hypotheses based on the knowledge obtained from our group and studies from other groups: (1) capacitation‐associated Na^+^ dynamics and NDSAT play a central role in the later events of mammalian sperm capacitation, and Em hyperpolarization upon capacitation is merely a result of such Na^+^ dynamics and (2) the physiological role of Slo3 is to maintain sperm morphology by regulating cell volume. The first hypothesis is summarized in Figure 2. During capacitation, NKA α4 activity is upregulated, whereas ENaC activity is downregulated via an action of CFTR. Both these events lead to a decrease in intracellular Na^+^, which in turn upregulates NDSAT, especially those regulating pH_i_. Consequently, an increase in pH_i_ occurs, which directly activates the CatSper Ca^2+^ channel, and VSP decreases PIP_2_ levels in the plasma membrane to downregulate NCX1. Consequently, sustained increases in intracellular Ca^2+^ levels are achieved, subsequently inducing AR and hyperactivation.

Further studies are necessary to validate the hypotheses proposed in this review and fully elucidate the complex and mysterious regulatory mechanisms of capacitation. Full elucidation of regulatory mechanisms will aid in developing improved ART, as Dr. Yanagimachi realized IVF not for medical purposes but for the purpose of basic science to investigate the mechanism of capacitation/fertilization.

## CONFLICT OF INTEREST STATEMENT

The authors declare no conflict of interest.
